# A conservation checklist of the herpetofauna of Morelos, with comparisons with adjoining states

**DOI:** 10.3897/zookeys.941.52011

**Published:** 2020-06-16

**Authors:** Julio A. Lemos-Espinal, Geoffrey R. Smith

**Affiliations:** 1 Laboratorio de Ecología-UBIPRO, FES Iztacala UNAM, Avenida los Barrios 1, Los Reyes Iztacala, Tlalnepantla, edo. de México, 54090, México FES Iztacala UNAM Tlalnepantla Mexico; 2 Department of Biology, Denison University, Granville, Ohio 43023, USA Denison University Granville United States of America

**Keywords:** amphibians, frogs, lizards, reptiles, salamanders, snakes, turtles

## Abstract

Despite being one of the smallest states in Mexico, the high diversity of habitats in Morelos has led to the development of a rich biota made up of a mixture of species typical of the Neovolcanic Axis and the Sierra Madre del Sur. However, recent expansion of cities in Morelos is likely to have consequences for the state’s herpetofauna. Here a checklist of the amphibians and reptiles of Morelos is provided with a summary of their conservation status and overlap with its neighboring states. Morelos is home to 139 species of amphibians and reptiles representing 32 families and 75 genera. Twenty-six of the 38 species of amphibians and 70 of the 101 species of reptiles that inhabit Morelos are endemic to Mexico. Fourteen species of amphibians and reptiles from Morelos are IUCN listed (i.e., Vulnerable, Near Threatened, or Endangered), 22 are placed in a protected category by SEMARNAT, and 41 are categorized as high risk by the EVS. The Tropical Deciduous Forest vegetation type hosts the greatest number of amphibian and reptile species in Morelos (84 species). Morelos shares the largest proportion of its herpetofauna with the State of Mexico (79.3%), Puebla (77.0%), and Guerrero (74.8%).

## Introduction

Morelos is one of the smallest states in Mexico; however, its high diversity of habitats has led to the development of a rich biota represented by a mixture of species typical of the Neovolcanic Axis and the Sierra Madre del Sur. The contrast in the habitat found in Morelos can be seen by the altitudinal gradient that occurs in its 4,961 km^2^ where altitude reaches a maximum of 5,380 m on the Popocatépetl Volcano, and a minimum of 800 m in the Río Amacuzac (INEGI 2017). In addition, Cuernavaca, the state capital located in northwestern Morelos, is known as the city of eternal spring for its pleasant and benign climate with little variation between seasons. This condition is not exclusive to Cuernavaca but prevails in most of the state, due to this and the proximity of Morelos to the metropolitan area of Mexico City, Morelos has become one of the favorite places for inhabitants of the Mexico City to spend weekends or vacations. This has also led to the growth of cities such as Cuernavaca, Jiutepec, Temixco, and Cuautla. As in other states, this population growth results in environmental degradation, including the clearing of forests, garbage generation, air and water pollution, and fragmentation of natural habitats. For example, in the dry forest of Morelos, the effects of grazing and timber harvesting have had significant effects on the vegetation of this habitat type resulting in fewer trees and a change in the herbaceous layer (de la O-Toris et al. 2012). In addition, many of the tropical dry forests and deciduous forests of Morelos are being lost to deforestation (García-Estrada et al. 2002; Navar et al. 2010). Indeed one study estimated that 60% of the original vegetation in Morelos had been removed by 1990 and only 19% was forested (Trejo and Dirzo 2000); however, the rate of deforestation appears to have slowed but not stopped, yet the forests have not recovered (Sotello-Caro et al. 2015). Such deforestation has increased habitat fragmentation with negative consequences for vertebrates (García-Estrada et al. 2002). Such changes in Morelos are likely to have consequences for the state’s fauna, including the amphibians and reptiles. It would be useful to develop an up-to-date inventory of such species as well as their conservation status as a first effort to understanding how to conserve and manage these species. Here we provide an up-to-date checklist of the amphibians and reptiles of Morelos and summarize their conservation status and overlap with species in its neighboring states.

### Physiographic characteristics of the state

**Figure 1.**
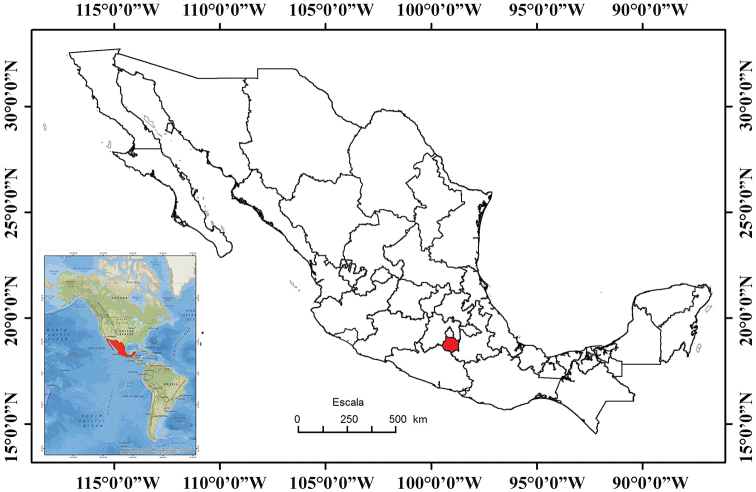
Map of Mexico with the state of Morelos shown in red (modified from INEGI 2018).

Morelos has an area of 4,961 km^2^ which represents only 0.2% of the total area of Mexico. Morelos is located in central-southern Mexico, between 19°07'54"N and 18°19'56"N and -98°37'58"W and -99°29'39"W. It is bordered by the State of Mexico and Mexico City to the north, Puebla to the east and southeast, Guerrero to the south and southwest, and the State of Mexico to the west (Fig. 1; INEGI 2017).

**Figure 2.**
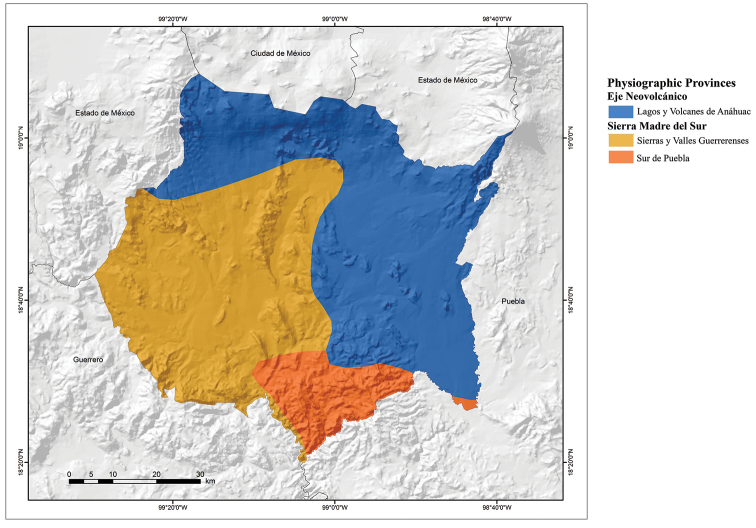
Physiographic provinces of the state of Morelos, Mexico (modified from Cervantes-Zamora et al. 1990).

Morelos contains portions of two physiographic provinces: the Neovolcanic Axis with one subprovince (Lagos y Volcanes de Anáhuac) and the Sierra Madre del Sur with two subprovinces (Sierras y Valles Guerrerenses and Sur de Puebla) (Fig. 2). The Neovolcanic Axis covers most of the state, from north to southeast, and the Sierra Madre del Sur covers the central and southwestern parts of the state (INEGI 2017). However, according to Aguilar (1990) the geological and physiographic characteristics of the northern part of Morelos are different from the plains of the east, so they should not be seen as the same province, and the southwestern part of the state is also not located within the Sierra Madre del Sur, but rather within the Balsas Basin. Thus, Morelos can be considered to include the physiographic provinces of the Neovolcanic Axis in the northern part of the state above 1,600 m asl, and the Balsas Basin found in the central and southern parts of the state (Contreras-MacBeath et al. 2006a).

**Figure 3.**
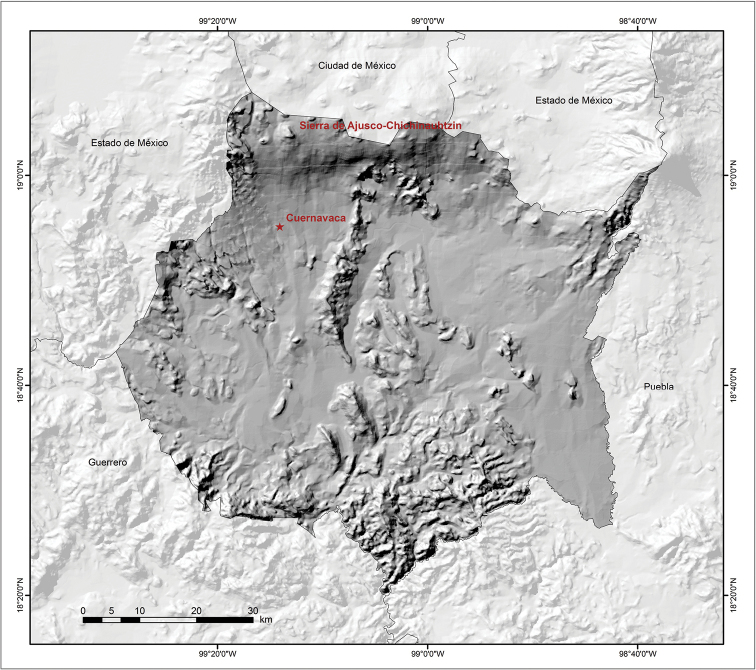
Topographical map of the state of Morelos, Mexico (INEGI 2009).

According to Monroy and Colin (1991), Morelos is divided into three ecological regions: the mountainous region of the north, the intermontane valley, and the mountainous region of the south (Fig. 3). The mountainous region of the north is characterized by temperate forest, both pine and oak, and some broadleaved associations. This region is found in the Neovolcanic Axis province. The intermontane valley is located in the central part of the state. Its natural resources have suffered a serious qualitative and quantitative decline due to the expansion of urban areas on the one hand and by pollution of the soil, water, and air on the other. In this region, most of the agricultural crops produced in the state are cultivated, although some patches of disturbed tropical deciduous forest can also be found here. The mountainous region of the south is in the Balsas Basin province, and is characterized by tropical deciduous forest, still preserved in some parts of the state (Contreras-MacBeath et al. 2006b).

**Figure 4.**
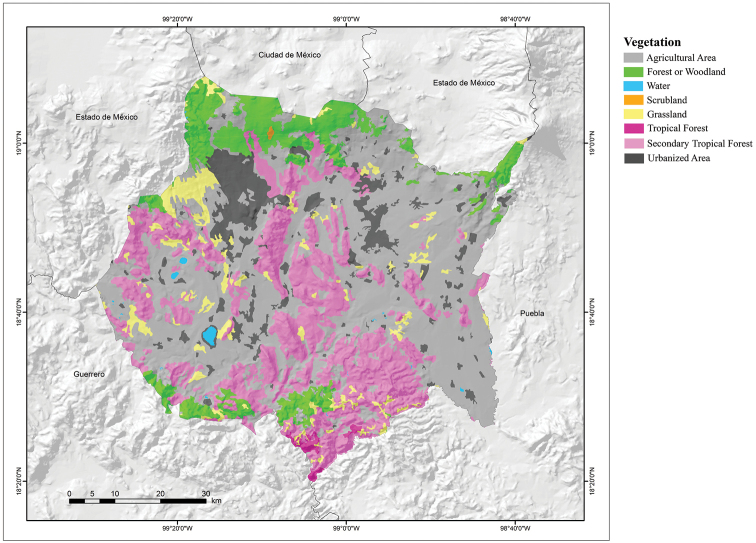
Vegetation map of the state of Morelos, Mexico (modified from Dirección General de Geografía – INEGI 2013).

The vegetation of Morelos is a product of the great heterogeneity of environmental conditions present in the state, and so it hosts a wide variety of vegetation types that for the purpose of this paper can be divided into three types, in addition to agricultural areas and areas devoid of vegetation (Fig. 4; INEGI 2017). These vegetation types are: Forests or Woodland (Oak, Pine-oak, Pine, and *Abies* Forests), Tropical Deciduous Forest, and Grasslands. The Woodlands can be divided in Coniferous Forest and Oak Forest. The Coniferous Forest is the most important of the forested areas that occupy the high elevations of the Neovolcanic Axis, mainly between 1,500 and 4,000 m a.s.l. This is a more or less dense community formed by a tree stratum that varies from 8 to 35 m high, with a broad floristic representation in the herbaceous and shrubby strata. This type of vegetation includes the following communities: a) pine-oak forest, b) pine forest, and c) *Abies* forest. The Oak Forest is distributed in the northern, southern, and southwestern parts of the state. Woodlands cover 11.45% of the total surface of Morelos (INEGI 2017). The Tropical Deciduous Forest develops in warm and semi-warm sub-humid climates. The largest area of this vegetation type is in the mountains of central and southern Morelos, between 900 and 1,600 m a.s.l. It is characterized by trees that lose their leaves almost completely during the dry season, between December and June, and produce their foliage and flowers in the rainy season. Tropical Deciduous Forest covers 27.61% of the total area of Morelos (INEGI 2017). The Grasslands are distributed in small areas, mainly in warm and subhumid semi-warm climates. They are located in flat areas or rolling hills. Alpine grassland is distributed in the highest mountain areas in northern Morelos, generally above 3,500 m a.s.l. (Contreras-MacBeath et al. 2006b). In Morelos Grasslands cover 4.29% of the surface area. The remaining 56.58% of the surface territory of Morelos is covered by agricultural areas and areas devoid of vegetation (INEGI 2017).

**Figure 5.**
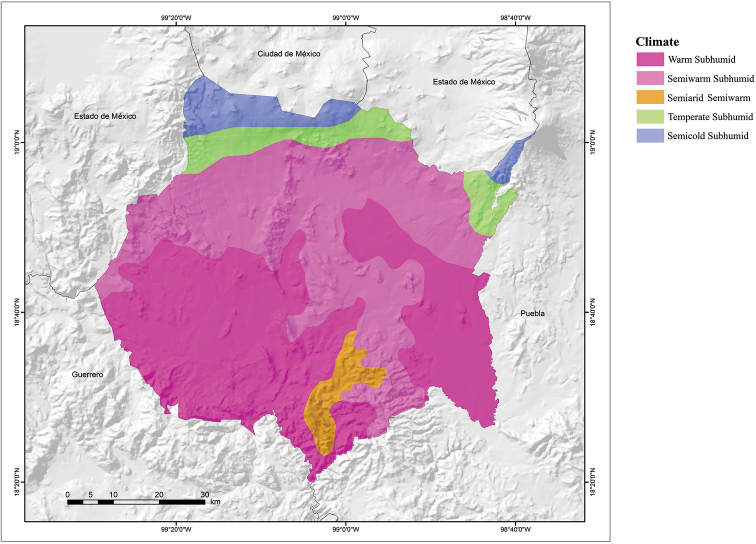
Climate map of the state of Morelos, Mexico (modified from García – Comisión Nacional para el Conocimiento y Uso de la Biodiversidad 1998).

Several climates (based on the classification of Köppen modified by García 1998) are found in Morelos (Fig. 5). Cold subhumid occurs in the highest parts of the Popocatépetl Volcano and to the northeast along the border with the State of Mexico and Mexico City and is characterized by an average annual temperature of less than 5 °C, with a high incidence of frost (Contreras-MacBeath et al. 2006a). According to the climatic units defined by Boyás (1992), this climate type only occurs in about 0.2% of the state. The semicold subhumid climate type is characterized by a long summer, with an average annual temperature between 5 and 12 °C and is located in the northern part of the state and south of the Sierra del Ajusco (Contreras-MacBeath et al. 2006a). According to the climatic units defined by Boyás (1992), this type of climate is found in 2% of the state. The temperate subhumid climate type has summer rains and is the wettest of the subhumid climates, with an average annual temperature between 5 and 12 °C, a long summer with the warmest months being April and May, and January the coldest. It is located in the northern part of the state (Contreras-MacBeath et al. 2006a). According to Boyás (1992 in Contreras-MacBeath et al. 2006a) this type of climate occurs in 10% of the state. The semihumid subhumid climate type is characterized by an average annual temperature between 18 and 22 °C, with summer rains and winter rains making up < 5% of the total annual rainfall (Contreras-MacBeath et al. 2006a). It is found in the northern part of Morelos and covers 16% of the state. The warm subhumid climate type is located throughout most of Morelos, but mainly in the central and southern parts. It is characterized by an average annual temperature > 22 °C, summer rains (from May to October), and a dry winter (< 5% of the total annual rainfall) (Contreras-MacBeath et al. 2006a). It covers 72% of the state area.

## Materials and methods

We compiled our list of amphibians and reptiles of Morelos from: (1) our field work; (2) a thorough examination of the available literature on amphibians and reptiles in the state; (3) amphibian and reptile records for Morelos in VertNet.org; and 4) amphibian and reptile records for Morelos in Servicio de Descarga de Ejemplares del Sistema Nacional de Información sobre Biodiversidad (SNIB-CONABIO), data bases Amphibians state of Morelos and Reptiles state of Morelos.

We follow Frost (2020) and AmphibiaWeb (2019) (http://amphibiaweb.org) for amphibian names and Uetz and Hošek (2019) for reptile names. We included species in the list if we could confirm records, either by direct observation or through documented museum records or vouchers. We do not include previously reported species for Morelos whose distribution is doubtful in the state because of a large gap between the currently known distributions of these species and the reports for Morelos. These species are: *Rana maculata* reported by Castro-Franco et al. (2006), which is distributed from eastern Oaxaca in the Isthmus of Tehuantepec, extending southeast to the central part of Nicaragua (Frost 2020); *Rana pustulosa* Boulenger, 1883 reported by Castro-Franco et al. (2006), which is distributed from southeastern Sonora and western Chihuahua extending south along the western slope of the Sierra Madre Occidental to Colima and Michoacán (Frost, 2020), the populations in Morelos previously considered as *R. pustulosa* are included in *R. zweifeli* (Hillis et al. 1984); *Rana vaillanti* Brocchi, 1877 reported by Castro-Franco et al. (2006) which is distributed from northern Veracruz and northern Oaxaca on the Atlantic slope and from southeastern Oaxaca and northwestern Chiapas on the Pacific slope, extending south through much of Central America, to southwestern Colombia and northwestern Ecuador (Frost 2020); and *Thamnophis sirtalis* (Linnaeus, 1758), reported by Castro-Franco and Bustos-Zagal (1994) as *T. dorsalis* (Baird & Girard, 1853) which is distributed from southeastern Alaska east to Nova Scotia and south across much of the United States, with isolated populations in Texas, New Mexico, and Chihuahua in northwestern Mexico (Fitch 1980). The southernmost record is reported in western Chihuahua, such that we consider it unlikely that this species occurs in Morelos. However, we did not examine any of the specimens used by Castro-Franco et al. (2006) to report these species, so we include them in the list of species that probably occur in Morelos (Table 2). On the other hand, there is a record of *Craugastor pygmaeus* (AMNH A-57809) collected in July 1953, by R. Ruibal at Tepozteco, and a record of *Eleutherodactylus verruculatus* (MVZ:Herp:36573) collected in July 1941, by Robert W. Storer, 12 mi S of Cuernavaca. We were unable to confirm the identity of these two specimens, so we do not include them in the species list for the state but we include them in the list of species that probably occurs in Morelos. Although we believe there is a high probability that *Ambystoma velasci* inhabits the eastern end of Morelos and there are seven records of this species for the state reported in Vertnet.org (MCZ A-24844-50: Museum of Comparative Zoology, Harvard University, Subset of data for VERTNET. Record ID: MCZ:Herp:A-24844. Source: http://digir.mcz.harvard.edu/ipt/resource.do?r=mcz_subset_for_vertnet) the locality reported in six of these records (24845-50) seems to place them in Puebla (circa 224-5 km from Mexico, Puebla, Mexico), and another (24844) is doubtful (circa 62 km S of Mexico, DF). Because of this, we decided not to include this species in the list of species presented here, but do include it in the list of species that probably occur in Morelos (see below).

We generated species accumulation curves for the total herpetofauna, amphibians, and reptiles using the year of the first recorded observation for each species. Such species accumulation curves can estimate potential species richness of amphibians and reptiles (see Raxworthy et al. 2012). In addition, we recorded the conservation status of each species based on the IUCN Red List 2019-2 (IUCN 2019); listing in SEMARNAT (2010); Environmental Vulnerability Scores from Wilson et al. (2013a, b) and Johnson et al. (2015).

The number of overlapping species with the three states and Mexico City that neighbor Morelos, was determined using recent check lists (Mexico City, Lemos-Espinal and Smith unpubl.; State of Mexico, Lemos-Espinal and Smith unpubl.; Guerrero, Palacios-Aguilar and Flores-Villela 2018; Puebla, Woolrich-Piña et al. 2017).

## Results and discussion

Morelos is home to 139 species of amphibians and reptiles representing 32 families (three of which are introduced: Gekkonidae, Typhlopidae, and Tryonicidae) and 75 genera (three of which are introduced: *Hemidactylus*, *Indotyphlops*, and *Apalone*) (Tables 1, 2). These include 38 species of amphibians (31 anurans [one introduced] and seven salamanders), and 101 reptiles (42 lizards [one introduced], 55 snakes [one introduced], and four turtles [one introduced]). The four introduced species are: the American Bullfrog (*Rana catesbeiana*), the Common House Gecko (*Hemidactylus frenatus*), the Brahminy Blindsnake (*Indotyphlops braminus*), and the Spiny Softshell (*Apalone spinifera*). The most speciose families of amphibians are Hylidae and Plethodontidae, whereas the most speciose families of reptiles are Phrynosomatidae and Colubridae (Tables 1, 2).

**Table 1.** Amphibians and reptiles of the state of Morelos with distributional and conservation status. Vegetation Type (VT): (1= Oak Forest; 2= Pine-oak Forest; 3= Pine Forest; 4= *Abies* Forest; 5= Tropical Deciduous Forest; 6= Grassland) according to Contreras-MacBeath et al. (2006b) and INEGI (2017). IUCN Status: (DD = Data Deficient; LC = Least Concern, VU = Vulnerable, NT = Near Threatened; EN = Endangered; CR = Critically Endangered; NE = not Evaluated) according to the IUCN Red List (The IUCN Red List of Threatened Species, Version 2019–2(www.iucnredlist.org; accessed 29November 2019); conservation status in Mexico according to SEMARNAT (2010) (CSM): (P = in danger of extinction, A = threatened, Pr = subject to special protection, NL – not listed); Environmental Vulnerability Score: (EVS – the higher the score the greater the vulnerability: low (L) vulnerability species (EVS of 3–9); medium (M) vulnerability species (EVS of 10–13); and high (H) vulnerability species (EVS of 14–20) from Wilson et al. (2013a,b) and Johnson et al. (2015); Global Distribution: 1= Endemic to Mexico; 2= Shared between the US and Mexico; 3= widely distributed from Mexico to Central or South America; 4= widely distributed from the US to Central or South America; IN = Introduced to Morelos. Date in which the first record appeared (1^st^); and Source of the first record.

	VT	IUCN	CSM	EVS	Global	1^st^	Source
**CLASS AMPHIBIA**							
**ORDER ANURA**							
**FAMILY BUFONIDAE**							
*Anaxyrus compactilis* (Wiegmann, 1833)	1,2,6	LC	NL	H (14)	1	1950	TCWC 6276
*Incilius marmoreus* (Wiegmann, 1833)	6	LC	NL	M (13)	1	1957	UAZ 11664
*Incilius occidentalis* (Camerano, 1879)	1,2,3,6	LC	NL	M (11)	1	1903	FMNH 17123
*Incilius perplexus* (Taylor, 1943)	5	EN	NL	M (11)	1	1936	FMNH 126950
*Rhinella horribilis* (Wiegmann, 1833)	5	LC	NL	L (3)	4	1901	FMNH 1620
**FAMILY CENTROLENIDAE**							
*Hyalinobatrachium fleischmanni* (Boettger, 1893)	5	LC	NL	M (10)	3	1999	CARUM 2742
**FAMILY CRAUGASTORIDAE**							
*Craugastor augusti* (Dugès, 1879)	1,2,3,6	LC	NL	L (8)	2	1972	LACM 106766
*Craugastor hobartsmithi* (Taylor, 1937)	1,5	EN	NL	H (15)	1	1975	MZFC 1089
*Craugastor rhodopis* (Cope, 1867)	1,5	VU	NL	H (14)	1	1930	FMNH 103253
*Craugastor rugulosus* (Cope, 1870)	3	LC	NL	M (13)	1	2004	Valenzuela-Galván et al. 2004a
**FAMILY ELEUTHERODACTYLIDAE**							
*Eleutherodactylus angustidigitorum* (Taylor, 1940)	1,2,3	VU	Pr	H (17)	1	1956	UCM 9223
*Eleutherodactylus maurus* Hedges, 1989	3	DD	Pr	H (17)	1	1953	AMNH A-57810
*Eleutherodactylus nitidus* (Peters, 1870)	5	LC	NL	M (12)	1	1938	FMNH 104455
**FAMILY HYLIDAE**							
*Dryophytes arenicolor* (Cope, 1886)	1,2,3,4,5,6	LC	NL	L (7)	2	1936	FMNH 99459
*Dryophytes eximius* (Baird, 1854)	1,2,3,4	LC	NL	M (10)	1	1932	FMNH 99712
*Dryophytes plicatus* (Brocchi, 1877)	1,2,3,4	LC	A	M (11)	1	1936	FMNH 27067
*Exerodonta smaragdina* (Taylor, 1940)	5	LC	Pr	M (12)	1	1943	Taylor 1943
*Sarcohyla bistincta* (Cope, 1877)	1,2,3	LC	Pr	L (9)	1	1936	CAS 87826
*Scinax staufferi* (Cope, 1865)	5	LC	NL	L (4)	3	1960	TCWC 16645
*Smilisca baudinii* (Duméril & Bibron, 1841)	5	LC	NL	L (3)	4	1949	TCWC 3576
*Tlalocohyla smithii* (Boulenger, 1902)	3,5	LC	NL	M (11)	1	1902	Boulenger, 1902
**FAMILY MICROHYLIDAE**							
*Gastrophryne olivacea* (Hallowell, 1856)	5	LC	Pr	L (9)	2	1938	FMNH 104397
*Hypopachus ustus* (Cope, 1866)	2,5	LC	Pr	L (7)	3	2004	Valenzuela-Galván et al. 2004b
*Hypopachus variolosus* (Cope, 1866)	2,5	LC	NL	L (4)	4	1936	FMNH 100572
**FAMILY PHYLLOMEDUSIDAE**							
*Agalychnis dacnicolor* (Cope, 1864)	5	LC	NL	M (13)	1	1905	USNM 57554
**FAMILY RANIDAE**							
*Rana catesbeiana* Shaw, 1802	**IN**	**IN**	**IN**	**IN**	**IN**	1971	ENCB 6943
*Rana forreri* Boulenger, 1883	5	LC	Pr	L (3)	3	1939	USNM 113856
*Rana montezumae* Baird, 1854	1,2,3,5	LC	Pr	M (13)	1	1983	KU KUH 195251
*Rana spectabilis* Hillis & Frost, 1985	1,2,3,5	LC	NL	M (12)	1	1938	FMNH 107767
*Rana zweifeli* Hillis, Frost & Webb, 1984	1,2,3,5	LC	NL	M (11)	1	1892	USNM 20165
**FAMILY SCAPHIOPODIDAE**							
*Spea multiplicata* (Cope, 1863)	1,2,5	LC	NL	L (3)	2	1930	FMNH 99013
**ORDER CAUDATA**							
**FAMILY AMBYSTOMATIDAE**							
*Ambystoma altamirani* Dugès, 1895	1,2,3,6	EN	A	M (13)	1	1939	USNM 116614
**FAMILY PLETHODONTIDAE**							
*Aquiloeurycea cephalica* (Cope, 1865)	1,2,3,4	NT	A	H (14)	1	1936	FMNH 114426
*Chiropterotriton orculus* (Cope, 1865)	1,2,3,4	VU	NL	H (18)	1	1902	Günther 1901
*Isthmura belli* (Gray, 1850)	1,2,3,4,6	VU	A	M (12)	1	1950	TCWC 6110
*Pseudoeurycea altamontana* (Taylor, 1939)	1,2,3,4	EN	Pr	H (17)	1	1939	Taylor 1939
*Pseudoeurycea leprosa* (Cope, 1869)	1,2,3,4	LC	A	H (16)	1	1933	FMNH 106158
*Pseudoeurycea tlilicxitl* Lara-Góngora, 2003	1,2,3,4	EN	NL	H (17)	1	1979	CNAR w/o #
**CLASS REPTILIA**							
**ORDER SQUAMATA**							
**SUBORDER LACERTILIA**							
**FAMILY ANGUIDAE**							
*Abronia deppii* (Wiegmann, 1828)	2	EN	A	H (16)	1	1981	MZFC 20215
*Barisia imbricata* (Wiegmann, 1828)	1,2,3,4,6	LC	Pr	H (14)	1	1936	FMNH 105770
*Barisia rudicollis* (Wiegmann, 1828)	1,2,3,5	EN	P	H (15)	1	1987	CARUM 508
*Gerrhonotus liocephalus* Wiegmann, 1828	5	LC	Pr	L (6)	1	1964	MSUM 6999
**FAMILY DACTYLOIDAE**							
*Anolis nebulosus* (Wiegmann, 1834)	1,2,5	LC	NL	M (13)	1	1892	USNM 20182
**FAMILY EUBLEPHARIDAE**							
*Coleonyx elegans* Gray, 1845	5	LC	A	L (9)	3	1950	TCWC 6548
**FAMILY GEKKONIDAE**							
*Hemidactylus frenatus* Duméril & Bribon, 1836	**IN**	**IN**	**IN**	**IN**	**IN**	2014	CARUM 2499
**FAMILY HELODERMATIDAE**							
*Heloderma horridum* (Wiegmann, 1829)	5	LC	A	M (11)	3	1932	FMNH 103953
**FAMILY IGUANIDAE**							
*Ctenosaura pectinata* (Wiegmann, 1834)	5	NE	A	H (15)	1	1939	CNAR 459
**FAMILIY PHRYNOSOMATIDAE**							
*Phrynosoma asio* Cope, 1864	5	LC	Pr	M (11)	1	2004	Castro-Franco and Bustos Zagal 2004
*Phrynosoma orbiculare* (Linnaeus, 1758)	1,2,3	LC	A	M (12)	1	1932	FMNH 102370
*Phrynosoma taurus* Bocourt, 1870	5	LC	A	M (12)	1	1998	CARUM 2622
*Sceloporus aeneus* Wiegmann, 1828	6	LC	NL	M (13)	1	1931	MCZ R-33914
*Sceloporus gadoviae* Boulenger, 1905	5	LC	NL	M (11)	1	1932	FMNH 32580
*Sceloporus grammicus* Wiegmann, 1828	1,2,3,4	LC	Pr	L (9)	1	1903	FMNH 1280
*Sceloporus horridus* Wiegmann, 1834	5	LC	NL	M (11)	1	1903	FMNH 1281
*Sceloporus melanorhinus* Bocourt, 1876	5	LC	NL	L (9)	3	1997	CARUM 2580
*Sceloporus mucronatus* Cope, 1885	1,2,3,4	LC	NL	M (13)	1	1970	BYU 36233
*Sceloporus ochoterenae* Smith, 1934	5	LC	NL	M (12)	1	1936	FMNH 33398
*Sceloporus palaciosi* Lara-Góngora, 1983	1,2,3,4	LC	NL	H (15)	1	1949	TCWC 3868
*Scelopours scalaris* Wiegmann, 1828	1,2,4,6	LC	NL	M (12)	1	1890	Günther 1901
*Sceloporus siniferus* Cope, 1870	5	LC	NL	M (11)	3	1977	CNAR 2375
*Sceloporus spinosus* Wiegmann, 1828	5	LC	NL	M (12)	1	1931	MCZ R-33912
*Sceloporus sugillatus* Smith, 1942	1,2,3	LC	NL	H (16)	1	1939	MCZ R-46762
*Sceloporus torquatus* Wiegmann, 1828	1,2,3	LC	NL	M (11)	1	1932	FMNH 32737
*Sceloporus utiformis* Cope, 1864	5	LC	NL	H (15)	1	2004	Castro-Franco and Bustos Zagal 2004
*Urosaurus bicarinatus* (Duméril, 1856)	5	LC	NL	M (12)	1	1899	CAS 3795
**FAMILY PHYLLODACTYLIDAE**							
*Phyllodactylus bordai* Taylor, 1942	1,5	LC	Pr	M (13)	1	1966	UAZ 55033
*Phyllodactylus lanei* Smith, 1935	1,5	LC	NL	H (15)	1	2008	Aréchaga-Ocampo et al. 2008
*Phyllodactylus tuberculosus* Wiegmann, 1834	1,5	LC	NL	L (8)	3	1997	CARUM 2385
**FAMILY SCINCIDAE**							
*Marisora brachypoda* (Taylor, 1956)	5	LC	NL	L (6)	3	1931	MCZ R-33689
*Plestiodon brevirostris* (Günther, 1860)	1,2,3	LC	NL	M (11)	1	1936	FMNH 114200
*Plestiodon copei* (Taylor, 1933)	1,2,3	LC	Pr	H (14)	1	1936	FMNH 114293
*Plestiodon indubitus* (Taylor, 1933)	1,2,3	NE	NL	H (15)	1	1933	Taylor, 1933
*Plestiodon lotus* Pavón-Vázquez, Nieto Montes de Oca, Mendoza-Hernández, Centenero-Alcalá, Santa Cruz-Padilla, & Jiménez-Arcos, 2017	1,5	NE	NL	NE	1	2017	Pavón-Vázquez et al. 2017
**FAMILY TEIIDAE**							
*Aspidoscelis communis* (Cope, 1878)	5	LC	Pr	H (14)	1	2004	Castro-Franco and Bustos Zagal 2004
*Aspidoscelis costatus* (Cope, 1878)	5	LC	Pr	M (11)	1	1906	NHMUK 1906.7.19.24–26
*Aspidoscelis deppii* (Wiegmann, 1834)	5	LC	NL	L (8)	3	1941	MVZ 36595
*Aspidoscelis guttatus* (Wiegmann, 1834)	5	LC	NL	M (12)	1	1980	CARUM 1255
*Aspidoscelis lineatissimus* (Cope, 1878)	5	LC	Pr	H (14)	1	1953	Davis and Smith 1953b
*Aspidoscelis sackii* (Wiegmann, 1834)	5	LC	NL	H (14)	1	1901	FMNH 1016
*Holcosus sinister* (Wiegmann, 1834)	5	NE	NL	M (13)	1	1956	USNM 139373
**SUBORDER SERPENTES**							
**FAMILY BOIDAE**							
*Boa sigma* Smith, 1943	5	NE	NL	H (15)	1	1949	TCWC 7401
**FAMILY COLUBRIDAE**							
*Conopsis biserialis* (Taylor & Smith, 1942)	1,2,3,4,6	LC	A	M (13)	1	1932	FMNH 126813
*Conopsis lineata* (Kennicott, 1859)	1,2,3,4,6	LC	NL	M (13)	1	1953	Davis and Smith 1953a
*Conopsis nasus* (Günther, 1858)	1,2,3,4,6	LC	NL	M (11)	1	1970	MCZ R-167269
*Drymarchon melanurus* (Duméril, Bibron & Duméril, 1854)	5	LC	NL	L (6)	3	1949	TCWC 4112
*Drymobius margaritiferus* (Schlegel, 1837)	5	LC	NL	L (6)	3	1903	USNM 46545
*Ficimia publia* (Cope, 1866)	5	LC	NL	L (9)	3	2004	Castro-Franco and Bustos Zagal 2004
*Lampropeltis polyzona* Cope, 1860	5	LC	NL	L (7)	1	1950	TCWC 7312
*Leptophis diplotropis* (Günther, 1872)	5	LC	A	H (14)	1	1953	Davis and Smith 1953a
*Masticophis mentovarius* (Duméril, Bibron & Duméril, 1854)	2,5	LC	A	L (6)	3	1938	FMNH 106202
*Mastigodryas melanolomus* (Cope, 1868)	5	LC	NL	L (6)	3	1974	CUMV R-0009974
*Oxybelis aeneus* (Wagler, 1824)	5	LC	NL	L (5)	4	1945	USNM 122059
*Pituophis deppei* (Dumeril, 1853)	1,2,3,5	LC	A	H (14)	1	1949	UMMZ 101931
*Pituophis lineaticollis* (Cope, 1861)	2,3	LC	NL	L (8)	3	1940	Taylor 1940a
*Pseudoficimia frontalis* (Cope, 1864)	5	LC	NL	M (13)	1	1938	FMNH 106367
*Salvadora bairdi* Jan & Sordelli, 1860	1,2,3,5	LC	Pr	H (15)	1	1953	Davis and Smith 1953a
*Salvadora mexicana* (Duméril, Bibron & Duméril, 1854)	5	LC	Pr	H (15)	1	1938	Taylor 1940
*Senticolis triaspis* (Cope, 1866)	1,2,5,6	LC	NL	L (6)	4	1860	CUMV R-0009673
*Sonora michoacanensis* (Dugès, 1884)	5	LC	NL	H (14)	1	1956	UCM 9080
*Tantilla bocourti* (Günther, 1895)	1,2,6	LC	NL	L (9)	1	1936	FMNH 111093
*Tantilla calamarina* Cope, 1866	1,2,3,6	LC	Pr	M (12)	1	1938	Taylor 1940
*Tantilla deppei* (Bocourt, 1883)	1,2,3,6	LC	A	M (13)	1	1949	TCWC 7350
*Trimorphodon biscutatus* (Duméril, Bibron & Duméril, 1854)	5	NE	NL	L (7)	3	1938	FMNH 106205
*Trimorphodon tau* Cope, 1870	5	LC	NL	M (13)	1	1938	FMNH 105287
**FAMILY DIPSADIDAE**							
*Coniophanes lateritius* Cope, 1862	3	DD	NL	M (13)	1	1945	Smith and Taylor 1945
*Coniophanes piceivittis* Cope, 1870	5	LC	NL	L (7)	3	1970	LSUMZ 73757
*Conophis vittatus* Peters, 1860	5	LC	NL	M (11)	3	1936	FMNH 104949
*Enulius flavitorques* (Cope, 1868)	5	LC	NL	L (5)	3	1939	Taylor, 1940a
*Hypsiglena torquata* (Günther, 1860)	5	LC	Pr	L (8)	1	1938	FMNH 105174
*Imantodes gemmistratus* (Cope, 1861)	5	LC	Pr	L (6)	3	1938	FMNH 125551
*Leptodeira maculata* (Hallowell, 1861)	5,6	LC	Pr	L (7)	1	2008	Aréchaga-Ocampo et al. 2008
*Leptodeira splendida* Günther, 1895	2,5	LC	NL	H (14)	1	1936	FMNH 105352
*Pseudoleptodeira latifasciata* (Günther, 1894)	5	LC	Pr	H (14)	1	1938	FMNH 99670
*Rhadinaea hesperia* Bailey, 1940	5	LC	Pr	M (10)	1	1892	USNM 20166
*Rhadinaea laureata* (Günther, 1868)	1,2,3	LC	NL	M (12)	1	1953	Davis and Smith 1953a
*Rhadinaea taeniata* (Peters, 1863)	1,2,3	LC	NL	M (13)	1	1932	USNM 110373
*Tropidodipsas zweifeli* (Liner & Wilson, 1970)	5	NE	Pr	H (16)	1	1966	AMNH R-115572
**FAMILY ELAPIDAE**							
*Micrurus laticollaris* Peters, 1870	5	LC	Pr	H (14)	1	1892	USNM 20167
*Micrurus tener* Baird & Girard, 1953	1,5	LC	NL	M (11)	2	1939	USNM 11334
**FAMILY LEPTOTYPHLOPIDAE**							
*Rena maxima* (Loveridge, 1932)	5	LC	NL	M (11)	1	1949	TCWC 4109
**FAMILY LOXOCEMIDAE**							
*Loxocemus bicolor* Cope, 1861	5	LC	Pr	M (10)	3	1938	Taylor 1940
**FAMILY NATRICIDAE**							
*Adelophis copei* Dugès, 1879	5	VU	Pr	H (15)	1	1940	USNM 110335
*Storeria storerioides* (Cope, 1866)	1,2,3	LC	NL	M (11)	1	1950	TCWC 7386
*Thamnophis cyrtopsis* (Kennicott, 1860)	1,2,3	LC	A	L (7)	4	1953	Davis and Smith 1953a
*Thamnophis eques* (Reuss, 1834)	1,2,3,4	LC	A	L (8)	2	1936	FMNH 106041
*Thamnophis scalaris* Cope, 1861	1,2,3,4	LC	A	H (14)	1	1936	FMNH 106285
**FAMILY TYPHLOPIDAE**							
*Indotyphlops braminus* (Daudin, 1803)	**IN**	**IN**	**IN**	**IN**	**IN**	1965	FMNH 154799
**FAMILY VIPERIDAE**							
*Agkistrodon bilineatus* Günther, 1863	5	NT	Pr	M (11)	3	1953	Davis and Smith 1953a
*Crotalus culminatus* Klauber, 1952	1,2,5	NE	NL	H (15)	1	1939	USNM 110610
*Crotalus molossus* Baird & Girard, 1853	1,2,3,4	LC	Pr	L (8)	2	1970	ENCB 6595
*Crotalus polystictus* (Cope, 1865)	1,2,3,4	LC	Pr	H (16)	1	1999	CNAR 19243
*Crotalus ravus* Cope, 1865	1,2,3	LC	A	H (14)	1	1953	Davis and Smith 1953a
*Crotalus tlaloci* Bryson, Linkem, Dorcas, Lathrop, Jones, Alvarado-Díaz, Grünwald & Murphy, 2014	1,2,3	NE	NL	H (16)	1	2014	Bryson et al. 2014
*Crotalus transversus* Taylor, 1944	4	LC	P	H (17)	1	1944	Taylor 1944
*Crotalus triseriatus* (Wagler, 1830)	1,2,3	LC	NL	H (16)	1	1949	TCWC 4131
**ORDER TESTUDINES**							
**FAMILY KINOSTERNIDAE**							
*Kinosternon hirtipes* (Wagler, 1830)	2,5	LC	Pr	M (10)	2	1892	USNM 20188
*Kinosternon integrum* LeConte, 1854	2,5	LC	Pr	M (11)	1	1936	UMMZ 80790
*Kinosternon scorpioides* (Linnaeus, 1766)	2,5	NE	Pr	M (10)	3	1964	TNHC 32286
** TRYONICIDAE **							
*Apalone spinifera* (Le Sueur, 1827)	**IN**	**IN**	**IN**	**IN**	**IN**	2004	Castro-Franco and Bustos Zagal 2004

**Table 2.** Summary of native species present in Morelos by Family, Order or Suborder, and Class. Status summary indicates the number of species found in each IUCN conservation status in the order DD, LC, VU, NT, EN, CE (see Table 1 for abbreviations; in some cases species have not been assigned a status by the IUCN and therefore these may not add up to the total number of species in a taxon). Mean EVS is the mean Environmental Vulnerability Score, scores ≥ 14are considered high vulnerability (Wilson et al. 2013a, b) and conservation status in Mexico according to SEMARNAT (2010) in the order NL, Pr, A, P (see Table 1for abbreviations).

**Scientific name**	**Numbers of genera**	**Nubmers of species**	**IUCN DD, LC, VU, NT, EN, CE**	**x̄ EVS**	**SEMARNAT NL, Pr, A, P**
**CLASS AMPHIBIA**					
**ORDER ANURA**	**17**	**30**	**1,25,2,0,2,0**	**10**	**21,8,1,0**
Bufonidae	3	5	0,4,0,0,1,0	10.4	5,0,0,0
Centrolenidae	1	1	0,1,0,0,0,0	10	1,0,0,0
Craugastoridae	1	4	0,2,1,0,1,0	12.5	4,0,0,0
Eleutherodactylidae	1	3	1,1,1,0,0,0	15.3	1,2,0,0
Hylidae	6	8	0,8,0,0,0,0	8.4	5,2,1,0
Microhylidae	2	3	0,3,0,0,0,0	6.7	1,2,0,0
Phyllomedusidae	1	1	0,1,0,0,0,0	13	1,0,0,0
Ranidae	1	4	0,4,0,0,0,0	9.8	2,2,0,0
Scaphiopodidae	1	1	0,1,0,0,0,0	3	1,0,0,0
**ORDER CAUDATA**	**5**	**7**	**0,1,2,1,3,0**	**15.3**	**2,1,4,0**
Ambystomatidae	1	1	0,0,0,0,1,0	13	0,0,1,0
Plethodontidae	4	6	0,1,2,1,2,0	15.7	2,1,3,0
**SUBTOTAL**	**22**	**37**	**1,26,4,1,5,0**	**11.0**	**23,9,5,0**
**CLASS REPTILIA**					
**ORDER SQUAMATA**	**49**	**95**	**1,80,1,1,2,0**	**11.6**	**54,24,15,2**
**SUBOR DERLACERTILIA**	**15**	**41**	**0,35,0,0,2,0**	**12.1**	**25,9,6,1**
Anguidae	3	4	0,2,0,0,2,0	12.8	0,2,1,1
Dactyloidae	1	1	0,1,0,0,0,0	13	1,0,0,0
Eublepharidae	1	1	0,1,0,0,0,0	9	0,0,1,0
Helodermatidae	1	1	0,1,0,0,0,0	11	0,0,1,0
Iguanidae	1	1	0,0,0,0,0,0	15	0,0,1,0
Phrynosomatidae	3	18	0,18,0,0,0,0	12.1	14,2,2,0
Phyllodactylidae	1	3	0,3,0,0,0,0	12	2,1,0,0
Scincidae	2	5	0,3,0,0,0,0	11.5	4,1,0,0
Teiidae	2	7	0,6,0,0,0,0	12.3	4,3,0,0
**SUBORDER SERPENTES**	**34**	**54**	**1,45,1,1,0,0**	**11.1**	**29,15,9,1**
Boidae	1	1	0,0,0,0,0,0	15	1,0,0,0
Colubridae	16	23	0,21,0,0,0,0	10.2	15,3,5,0
Dipsadidae	9	13	1,11,0,0,0,0	10.5	7,6,0,0
Elapidae	1	2	0,2,0,0,0,0	12.5	1,1,0,0
Leptotyphlopidae	1	1	0,1,0,0,0,0	11	1,0,0,0
Loxocemidae	1	1	0,1,0,0,0,0	10	0,1,0,0
Natricidae	3	6	0,4,1,0,0,0	11	2,1,3,0
Viperidae	2	8	0,5,0,1,0,0	14.1	3,3,1,1
**ORDER TESTUDINES**	**1**	**3**	**0,2,0,0,0,0**	**10.3**	**0,3,0,0**
Kinosternidae	1	3	0,2,0,0,0,0	10.3	0,3,0,0
**SUBTOTAL**	**50**	**98**	**1,82,1,1,2,0**	**11.5**	**54,27,15,2**
**TOTAL**	**72**	**135**	**2,108,5,2,7,0**	**11.4**	**77,36,20,2**

**Figure 6.**
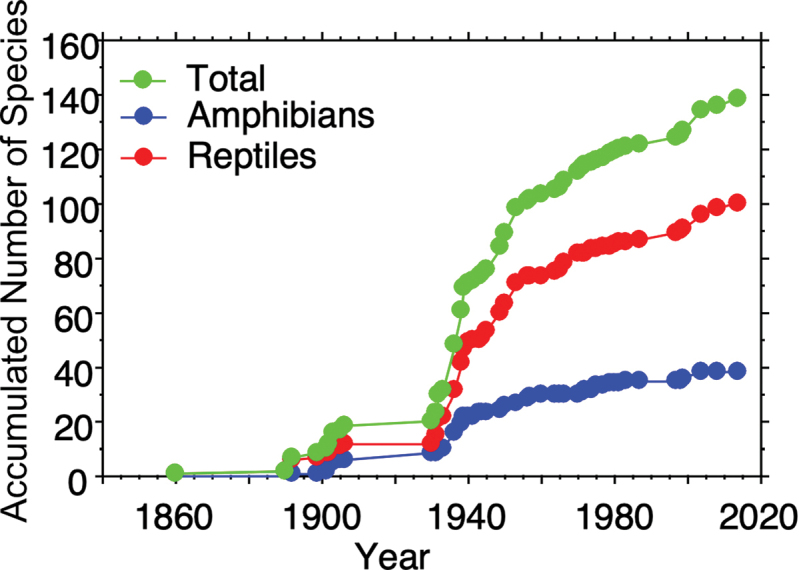
Species accumulation curves for total herpetofauna, amphibians, and reptiles of Morelos, Mexico.

We compiled a list of 21 species (eight amphibians, 13 reptiles) that we believe potentially occur in Morelos (Table 3). We created this list from species that are distributed near the border with Morelos in southern Mexico City, west-central State of Mexico, northern Guerrero, and southwestern Puebla. The distributional records we used to create this list were found in Vertnet.org and Sistema Nacional de Información sobre Biodiversidad (SNIB-CONABIO) for the three neighboring states and Mexico City. We are convinced that as more herpetological work is done near borders with these neighboring states, these “likely to occur” species, will be recorded for Morelos. Indeed, the species accumulation curves suggest that our checklist is likely to underestimate the number of species present in Morelos, especially for reptiles (Fig. 6). In particular, there was a relatively steady increase in species documented in Morelos throughout the 20^th^ Century, and while the rate of species being added to the known herpetofauna in Morelos has slowed more recently, particularly for amphibians, it has continued. We therefore predict that more species will be added to our list as more survey and systematic work in the state and region are completed.

**Table 3.** List of amphibian and reptile species that potentially occur in Morelos

	Likely to occur in:
CLASS AMPHIBIA	
**ORDER ANURA**	
**FAMILY CRAUGASTORIDAE**	
*Craugastor pygmaeus* (Taylor, 1937)	recorded at Tepozteco (AMNH A-57809)
**FAMILY ELEUTHERODACTYLIDAE**	
*Eleutherodactylus verruculatus* (Peters, 1870)	recorded at 12mi S of Cuernavaca (MVZ 36573)
**FAMILY LEPTODACTYLIDAE**	
*Leptodactylus fragilis* (Brocchi, 1877)	western and/or eastern Morelos
*Leptodactylus melanonotus* (Hallowell, 1861)	western, southern, and/or eastern Morelos
**FAMILY RANIDAE**	
*Rana maculata* Brocchi, 1877	reported by Castro-Franco et al. (2006)
*Rana pustulosa* Boulenger, 1883	reported by Castro-Franco et al. (2006)
*Rana vaillanti* Brocchi, 1877	reported by Castro-Franco et al. (2006)
**ORDER CAUDATA**	
**FAMILY AMBYSTOMATIDAE**	
*Ambystoma velasci* Dugès, 1888	eastern Morelos
**CLASS REPTILIA**	
**SUBORDER LACERTILIA**	
**FAMILY PHRYNOSOMATIDAE**	
*Sceloporus anahuacus* Lara-Góngora, 1983	northern Morelos
*Sceloporus pyrocephalus* Cope, 1864	western Morelos
**FAMILY SCINCIDAE**	
*Plestiodon lynxe* (Wiegmann, 1834)	northern and/or western Morelos
**SUBORDER SERPENTES**	
**FAMILY COLUBRIDAE**	
*Tantilla rubra* Cope, 1875	eastern Morelos
**FAMILY DIPSADIDAE**	
*Diadophis punctatus* (Linnaeus, 1766)	northern Morelos
*Geophis bicolor* Günther, 1868	northern Morelos
*Geophis petersii* Boulenger, 1894	northern Morelos
**FAMILY ELAPIDAE**	
*Micrurus browni* Schmidt & Smith, 1943	northwestern Morelos
**FAMILY NATRICIDAE**	
*Thamnophis sirtalis* (Linnaeus, 1758)	reported as *T. dorsalis* by Castro-Franco and Bustos-Zagal (1994)
*Thamnophis melanogaster* (Wiegmann, 1830)	northern Morelos
*Thamnophis pulchrilatus* (Cope, 1885)	northern Morelos
*Thamnohis scaliger* (Jan, 1863)	northern Morelos
**ORDER TESTUDINES**	
**FAMILY EMYDIDAE**	
*Trachemys venusta* (Gray, 1855)	eastern Morelos

### General distribution

Nineteen of the 31 species of anuran that inhabit Morelos are endemic to Mexico. Four of the twelve non-endemic species to Mexico are distributed in the United States and Mexico, another four range from Mexico to Central America, three more are distributed from the United States to Central America or South America, and one is introduced to Morelos. All seven species of salamanders that inhabit Morelos are endemic to Mexico.

Thirty-three of the 42 species of lizards that inhabit Morelos are endemic to Mexico. Of the nine species of lizards not endemic to Mexico, only one is found in the US and Mexico (*Sceloporus grammicus*), another seven range from Mexico to Central America, and the remaining species is introduced to Morelos. Thirty-five of the 55 species of snakes that inhabit Morelos are endemic to Mexico. Three of the 20 non-endemic species to Mexico are found in the US and Mexico, 13 are distributed from Mexico to Central America or South America, three occur from the US to Central America or South America, and one is introduced to Morelos. One of the four species of turtles that inhabit Morelos is endemic to Mexico, one occurs in the US and Mexico, one is distributed from Mexico to South America, and one is introduced to Morelos.

### Conservation status

**Figure 7.**
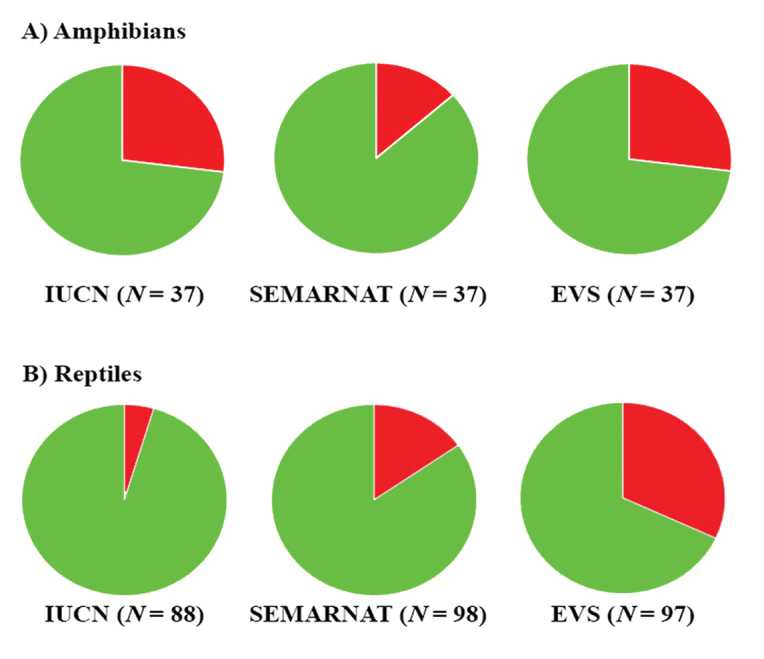
Percent of **A** amphibians and **B** reptiles listed in protected categories on the IUCN Red List and SEMARNAT. Green is percentage in Data Deficient and Least Concern (IUCN); Not Listed and Subject to Special Protection (we regarded the category of Subject to Special Protection in SEMARNAT equivalent to Least Concern in IUCN) (SEMARNAT). Red is percentage in protected categories. N is the number of species assessed by each agency.

A total of 14 (= 11.2% [14/125]) species of amphibians and reptiles is IUCN listed (i.e., Vulnerable, Near Threatened, or Endangered), 22 (= 16.3% [22/135]) are placed in a protected category (excluding NL and Pr, this last category is equivalent to the LC category of IUCN) by SEMARNAT and 41 species (= 30.6% [41/134]) are categorized as high risk by the EVS (Fig. 7; Table 3). For amphibians, 27.0% [10/37] are IUCN listed, 13.5% (5/37) are protected by SEMARNAT, and 27.0% [10/37] are at high risk according to the EVS (Fig. 7; Table 3). For reptiles, 4.5% [4/88] are listed by the IUCN, 17.3% [17/98] are protected by SEMARNAT, and 32.0% [31/97] are at high risk according to the EVS (Fig. 7; Table 3). These results suggest that both amphibians and reptiles in the state of Morelos are considered to have relatively low conservation status at global (IUCN) and local (SEMARNAT and EVS) scales. However, although in general the number of species considered in high risk by the EVS is relatively low, this number is greater than that considered in categories of conservation concern by IUCN and SEMARNAT, which is an indicator that the most reliable system to categorize species with some conservation status is the EVS. Although the IUCN evaluation is global, in general it should reflect the conservation status faithfully for the Morelos herpetofauna since 71.1% (96/135) of its species are endemic to the country, so the global evaluation in this case is based in more local or regional evaluations. On the other hand, the Mexican government (SEMARNAT) released a new update in 2019 but it does not appear conservation statuses have been reevaluated since 2010 because all Morelos statuses for amphibians and reptiles have remained the same, so although it is a local evaluation, it might not reflect the current conservation status of the species. The best example of this is the differences that exist in these three evaluation systems in two of the Morelos salamanders: *Chiropterotriton orculus* is regarded as Vulnerable (VU) by the IUCN, is not considered in any protection category by SEMARNAT, and has a value of 18 (high risk) according to the EVS; and *Pseudoeurycea tlilicxitl* is considered Endangered (EN) by the IUCN, is not considered in any protection category by SEMARNAT, and has a value of 17 (high risk) according to the EVS. Similar differences occur in several species of Morelos herpetofauna, suggesting an updated assessment of the conservation of Mexican amphibians and reptiles by SEMARNAT is sorely needed.

### Habitat types

The vegetation type that hosts the greatest number of amphibian and reptile species in Morelos is the Tropical Deciduous Forest (Table 4), which represent 63.0% (85/135) of the total number of species. However, it is also the vegetation type that has the lowest percentage of species protected by the IUCN or SEMARNAT, and except for the Grassland, it is also the type of vegetation with the lowest number of species categorized as high risk by the EVS (Table 5). The vegetation type of Morelos with the second richest herpetofauna is Pine-oak Forest with 62 species (45.9% of the species richness of Morelos), followed by Oak Forest with 60 species (21 amphibians, 39 reptiles: 44.4% of the species richness of Morelos) (Table 4). Although these two vegetation types house fewer species than the Tropical Deciduous Forest, they have much higher percentages of species protected by the IUCN and SEMARNAT or categorized as high risk by the EVS. In fact, if Grassland is excluded, the two vegetation types with the lowest numbers of amphibian and reptile species, the Pine and *Abies* Forests, are also the two vegetation types with the highest percentages of species protected by the IUCN and SEMARNAT or categorized as high risk by the EVS (Table 5). The small number of species inhabiting the Pine and *Abies* Forest is due to the small areas that these two vegetation types occupy in the state, according to SARH (1994), in Morelos, the Pine Forest occupies 80.7 km^2^ (1.7% of the state area), and the *Abies* Forest occupies 22.7 km^2^ (0.5% of the state area), and both are distributed mainly in the northern part of the state.

**Table 4.** Number of amphibian and reptile species in each vegetation type of Morelos

	**Oak Forest**	**Pine-oak Forest**	**Pine Forest**	***Abies* Forest**	**Tropical Deciduous Forest**	**Grassland**
Amphibians	21	21	20	9	20	7
Reptiles	39	41	31	13	65	11
Total	60	62	51	22	85	18

**Table 5.** Number of amphibian and reptile species in each vegetation type of Morelos listed and protected in the IUCN Red List or SEMARNAT list, or with a high EVS. Numbers in parenthesis represent the number of species not evaluated by the IUCN.

	IUCN		SEMARNAT		EVS	
**Amphibians**						
Oak Forest	9	12	5	16	9	12
Pine-oak Forest	7	14	5	16	7	14
Pine Forest	7	13	5	15	7	13
*Abies* Forest	5	4	4	5	5	4
Tropical Deciduous Forest	3	17	–	20	2	18
Grassland	2	5	2	5	1	6
**Reptiles**						
Oak Forest	1	34(3)	9	30	15	23
Pine-oak Forest	2	35(4)	11	30	16	25
Pine Forest	1	28(2)	9	22	13	18
*Abies* Forest	–	13	4	9	5	8
Tropical Deciduous Forest	3	52(9)	9	57	19	45
Grassland	–	11	2	9	1	10

### Comparison with neighboring states

Morelos shares the largest proportion of its amphibian and reptile species with the State of Mexico; however, this percentage is very similar to that of the species shared with Puebla and Guerrero (Table 6). These high percentages of shared species are due to a combination of the extent of the borders between Morelos and each of these three states, and the territorial size of each of them. Although the State of Mexico is smaller than Puebla and Guerrero, it surrounds almost the entire northern half of Morelos, especially if one considers that Mexico City is essentially a part of the State of Mexico from a herpetofaunal point of view (i.e., one could consider Mexico City as an extension of the State of Mexico in this context) (Fig. 1). This large contact area likely results in a high percentage of shared species. For example, all species of salamanders that inhabit Morelos, are also found in the State of Mexico, and five of the nine families of anurans that inhabit Morelos are fully shared with the State of Mexico. Only five species of Morelos anurans do not inhabit the State of Mexico, resulting in the highest percentage of amphibian species shared in the region and the highest percentage of shared herpetofauna. However, Morelos shares a similar proportion of reptile species with Guerrero, Puebla and the State of Mexico (Table 6). Thus, Morelos shares an almost equal proportion of amphibian and reptile species with these three states, and an explanation for the difference in the species shared with each of them is found in the large number of salamanders that Morelos shares with the State of Mexico. This is due to the fact that these two states share the temperate habitats of northern Morelos, which host this unique assortment of salamander species, since the number of reptile species that Morelos shares with each of these three states is virtually the same, regardless of size of the state.

**Table 6.** Summary of the numbers of species shared between Morelos and neighboring Mexican states (not including introduced species). The percent of Morelos species shared by a neighboring state are given in parentheses. Total refers to the total number of species found in Morelos and four neighboring states (i.e., regional species pool) and the number in parentheses in this column is the percent of the regional species pool found in Morelos. – indicates either Morelos or the neighboring state has no species in the taxonomic group, or none of that specific taxon is shared between the states, thus no value for shared species is provided.

TAXON	Morelos	Mexico	Puebla	Guerrero	Mexico City	TOTAL
**CLASS AMPHIBIA**	**37**	**32(86.5)**	**28(75.7)**	**24(64.9)**	**14(37.8)**	**150(24.7)**
**ORDER ANURA**	**30**	**25(83.3)**	**25(83.3)**	**22(73.3)**	**7(23.3)**	**100(30.0)**
Bufonidae	5	5(100)	5(100)	4(80.0)	1(20.0)	10(50.0)
Centrolenidae	1	–	1(100)	1(100)		1(100)
Craugastoridae	4	3(75.0)	2(50.0)	3(75.0)	1(25.0)	16(25.0)
Eleutherodactylidae	3	3(100.0)	1(33.3)	1(33.3)	–	11(27.3)
Hylidae	8	7(87.5)	8(100)	7(87.5)	3(37.5)	40(20.0)
Leptodactylidae	–	–	–	–	–	2(0)
Microhylidae	3	1(33.3)	2(66.7)	2(66.7)	–	3(100)
Phyllomedusidae	1	1(100)	1(100)	1(100)	–	3(33.3)
Ranidae	4	4(100)	3(75.0)	2(50.0)	1(25.0)	12(33.3)
Rhinophrynidae	–	–	–	–	–	1(0)
Scaphiopodidae	1	1(100)	1(100)	1(100)	1(100)	1(100)
**ORDER CAUDATA**	**7**	**7(100)**	**3(42.9)**	**2(28.6)**	**7(100)**	**49(14.3)**
Ambystomatidae	1	1(100)	–	–	1(100)	10(10.0)
Plethodontidae	6	6(100)	3(50.0)	2(33.3)	6(100)	38(15.8)
Salamandridae	–	–	–	–	–	1(0)
**ORDER GYMNOPHIONA**	–	–	–	–	–	**1(0)**
Caecilidae	–	–	–	–	–	1(0)
**CLASS REPTILIA**	**98**	**75(76.5)**	**76(77.6)**	**77(78.6)**	**35(35.7)**	**294(33.3)**
**ORDER CROCODYLIA**	–	–	–	–	–	**1(0)**
Crocodylidae	–	–	–	–	–	1(0)
**ORDER SQUAMATA**	**95**	**73(76.8)**	**75(78.9)**	**76(80.0)**	**33(34.7)**	**280(33.9)**
**SUBORDEN AMPHISBAENIA**	–	–	–	–	–	**2(0)**
Bipedidae	–	–	–	–	–	2(0)
**SUBORDER LACERTILIA**	**41**	**30(73.2)**	**29(70.7)**	**35(85.4)**	**12(29.3)**	**118(34.7)**
Anguidae	4	4(100)	2(50.0)	3(75.0)	1(25.0)	11(36.4)
Corytophanidae	–	–	–	–	–	3(0)
Dactyloidae	1	1(100)	–	1(100)	–	18(5.6)
Diploglossidae	–	–	–	–	–	2(0)
Eublepharidae	1	–	1(100)	1(100)	–	1(100)
Helodermatidae	1	1(100)	1(100)	1(100)	–	1(100)
Iguanidae	1	1(100)	1(100)	1(100)	–	4(25.0)
Phrynosomatidae	18	14(77.8)	15(83.3)	15(83.3)	9(50.0)	36(50.0)
Phyllodactylidae	3	1(33.3)	1(33.3)	3(100)	–	5(60.0)
Scincidae	5	4(80.0)	4(80.0)	4(80.0)	2(40.0)	15(33.3)
Teiidae	7	4(57.1)	4(57.1)	6(85.7)	–	12(58.3)
Xantusidae	–	–	–	–	–	5(0)
Xenosauridae	–	–	–	–	–	5(0)
**SUBORDER SERPENTES**	**54**	**43(79.6)**	**46(85.2)**	**41(75.9)**	**21(38.9)**	**160(33.8)**
Boidae	1	1(100)	1(100)	–	–	2(50.0)
Colubridae	23	20(87.0)	22(95.7)	20(87.0)	9(39.1)	41(56.1)
Dipsadidae	13	8(61.5)	10(76.9)	11(84.6)	2(15.4)	62(21.0)
Elapidae	2	2(100)	2(100)	1(50.0)	1(50.0)	10(20.0)
Leptotyphlopidae	1	1(100)	1(100)	1(100)	–	6(16.7)
Loxocemidae	1	–	–	1(100)	–	1(100)
Natricidae	5	4(80.0)	4(80.0)	3(60.0)	4(80.0)	16(31.3)
Typhlopidae	–	–	–	–	–	1(0)
Viperidae	8	7(87.5)	6(75.0)	4(50.0)	5(62.5)	21(38.1)
**ORDER TESTUDINES**	**3**	**2(66.7)**	**1(33.3)**	**1(33.3)**	**2(66.7)**	**13(23.1)**
Cheloniidae	–	–	–	–	–	3(0)
Dermochelyidae	–	–	–	–	–	1(0)
Emydidae	–	–	–	–	–	2(0)
Geoemydidae	–	–	–	–	–	2(0)
Kinosternidae	3	2(66.7)	1(33.3)	1(33.3)	2(66.7)	5(60.0)
**TOTAL**	**135**	**107(79.3)**	**104(77.0)**	**101(74.8)**	**49(36.3)**	**444(30.4)**

## Appendix 1

Museum collections included in the VertNet.org database records of Morelos amphibians and reptiles that house specimens of the first record of a species in Morelos.

**AMNH** Collection of Herpetology, Herpetology Department, American Museum of Natural History

**NHMUK** Zoological Collection, British Museum of Natural History, London

**BYU** Herpetology Collection, Monte L. Bean Museum, Brigham Young University

**CNAR** Colección Nacional de Anfibios y Reptiles, Instituto de Biología, Universidad Nacional Autónoma de México

**CUMV** Amphibian and Reptile Collection, Cornell University Museum of Vertebrates

**CARUM** Colección de Anfibios y Reptiles, Universidad Autónoma del Estado de Morelos

**CAS** Collection of Herpetology, Herpetology Department, California Academy of Sciences

**ENCB** Escuela Nacional de Ciencias Biológicas, Instituto Politécnico Nacional

**FMNH** Division of Amphibians and Reptiles, Field Museum of Natural History

**KU KUH** Herpetology Collection, University of Kansas Biodiversity Institute

**LACM** Collection of Herpetology, Herpetology Section, Natural History Museum of Los Angeles County

**LSUMZ** Collection of Reptiles and Amphibians, Louisiana State University Museum of Natural Science

**MCZ** Collection of Herpetology, Museum of Comparative Zoology, Harvard University Cambridge

**MSUM** Ichthyology and Herpetology Collections, Michigan State University Museum

**MVZ** Herpetological Collection, Museum of Vertebrate Zoology at Berkeley

**MZFC** Colección Herpetológica, Museo de Zoología Alfonso L. Herrera, Facultad de Ciencias, UNAM

**TCWC** Collection of Herpetology, Texas Cooperative Wildlife Collection, Texas & University

**TNHC** Collection of Herpetology, Texas Natural History Collection, University of Texas Austin

**UAZ** Amphibians and Reptiles Collections, University of Arizona

**UCM** Collection of Herpetology, University of Colorado Museum

**UMMZ** Collection of Herpetology, Museum of Zoology, University of Michigan Ann Arbor

**USNM** Collection of Herpetology, Department of Vertebrate Zoology, National Museum of Natural History, Smithsonian Institution
